# 4-Carb­oxy­anilinium chloride

**DOI:** 10.1107/S1600536811046721

**Published:** 2011-11-09

**Authors:** Li-Jun Han, Shu-Ping Yang, Xin Tao, Yuan-Feng Ma

**Affiliations:** a Department of Mathematics and Science, Huaihai Institute of Technology, Lianyungang 222005, People’s Republic of China; b Department of Chemical Engineering, Huaihai Institute of Technology, Lianyungang 222005, People’s Republic of China

## Abstract

In the title salt, C_7_H_8_NO_2_^+^·Cl^−^, the cation and anion are linked by an O—H⋯Cl hydrogen bond. The three-dimensional crystal structure is stabilized by N—H⋯O and N—H⋯Cl hydrogen bonds.

## Related literature

For related structures, see: Athimoolam & Natarajan (2007 ▶); Gracin & Fischer (2005 ▶).
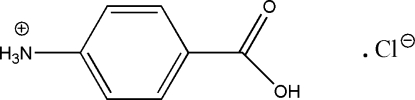


## Experimental

### 

#### Crystal data

C_7_H_8_NO_2_^+^·Cl^−^
*M* *_r_* = 173.59
Monoclinic, 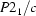

*a* = 5.601 (5) Å
*b* = 8.269 (5) Å
*c* = 17.118 (5) Å
β = 96.371 (5)°
*V* = 787.9 (9) Å^3^
*Z* = 4
Mo *K*α radiation
μ = 0.43 mm^−1^
*T* = 298 K
0.50 × 0.40 × 0.30 mm

#### Data collection

Bruker APEXII CCD area-detector diffractometer
Absorption correction: multi-scan (*SADABS*; Bruker, 2008 ▶) *T*_min_ = 0.814, *T*_max_ = 0.882
4205 measured reflections
1299 independent reflections
1210 reflections with *I* > 2σ(*I*)
*R*_int_ = 0.030

#### Refinement

*R*[*F* ^2^ > 2σ(*F* ^2^)] = 0.054
*wR*(*F* ^2^) = 0.192
*S* = 1.26
1299 reflections
133 parameters
All H-atom parameters refined
Δρ_max_ = 0.49 e Å^−3^
Δρ_min_ = −0.34 e Å^−3^

### 

Data collection: *APEX2* (Bruker, 2008 ▶); cell refinement: *SAINT* (Bruker, 2008 ▶); data reduction: *SAINT*; program(s) used to solve structure: *SHELXS97* (Sheldrick, 2008 ▶); program(s) used to refine structure: *SHELXL97* (Sheldrick, 2008 ▶); molecular graphics: *DIAMOND* (Brandenburg & Berndt, 1999 ▶); software used to prepare material for publication: *SHELXL97*.

## Supplementary Material

Crystal structure: contains datablock(s) I, global. DOI: 10.1107/S1600536811046721/wn2458sup1.cif

Structure factors: contains datablock(s) I. DOI: 10.1107/S1600536811046721/wn2458Isup2.hkl

Supplementary material file. DOI: 10.1107/S1600536811046721/wn2458Isup3.cml

Additional supplementary materials:  crystallographic information; 3D view; checkCIF report

## Figures and Tables

**Table 1:** Hydrogen-bond geometry (Å, °)

*D*—H⋯*A*	*D*—H	H⋯*A*	*D*⋯*A*	*D*—H⋯*A*
O1—H1⋯Cl1	0.99 (8)	2.10 (8)	3.059 (4)	164 (6)
N1—H1*A*⋯Cl1^i^	0.85 (6)	2.33 (6)	3.154 (6)	165 (5)
N1—H1*B*⋯O2^ii^	0.88 (9)	2.05 (8)	2.823 (6)	145 (7)
N1—H1*B*⋯Cl1^iii^	0.88 (9)	2.70 (9)	3.289 (5)	125 (6)
N1—H1*C*⋯Cl1^ii^	0.96 (8)	2.26 (8)	3.215 (5)	172 (6)

Symmetry codes: (i) 
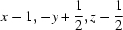
; (ii) 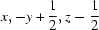; (iii) 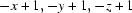.

**Fig. 1.**
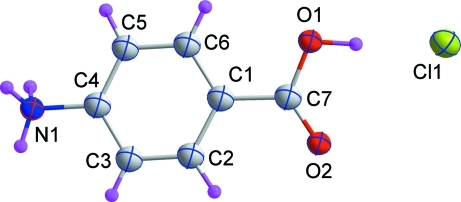
The asymmetric unit of the title structure. Displacement ellipsoids are drawn at the 50% probability level. Hydrogen atoms are shown as spheres of arbitrary radius.

**Fig. 2.**
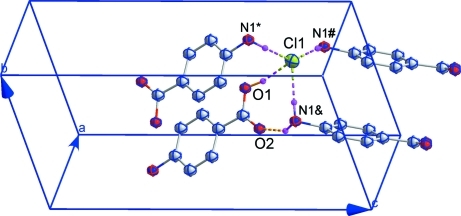
Part of the crystal structure, with hydrogen bonds shown as dashed lines. For clarity, H atoms not involved in the hydrogen bonds have been omitted. [Symmetry code:(*).1 - x, 1 - y, 1 - z (#). 1 + x, 1/2 - y, 1/2 + z,(&). x, 1/2 - y, 1/2 + z].

## supplementary crystallographic information

### Comment

We intended to prepare a cerium(III) complex of *p*-aminobenzoic acid. However, we obtained crystals of the title salt, and we report here its crystal structure.

In the title salt, the asymmetric unit consists of one *p*-aminobenzoic acid cation and one chloride anion (Fig. 1).

The amine group is protonated and the C4—N1 bond length is 1.471 (7) Å. In the crystal structure of 4-carboxyanilinium(2*R*, 3*R*)-tartrate (Athimoolam & Natarajan, 2007) the amine group is also protonated and the values of the corresponding C—N bond lengths are 1.464 (6) Å and 1.476 (5) Å.

In the crystal structures of the α-polymorph of *p*-aminobenzoic acid (Athimoolam & Natarajan, 2007) and β-polymorph of *p*-aminobenzoic acid (Gracin & Fischer, 2005) the amino group is not protonated. For the α-polymorph the C—N distance is 1.372 (5) Å; for the β-polymorph the distance is 1.408 (3) Å.

The hydrogen bonds listed in Table 1 result in a crystal structure generated by inversion and glide symmetry (Fig. 2).

### Experimental

To a solution containing *p*-aminobenzoic acid (1.37 g, 10 mmol) in ethanol (30 ml), a solution of cerium(III) chloride (1.24 g, 5 mmol) in methanol (15 ml) was added with stirring for 2 h at 323 K, and then the solution was filtered. Colourless crystals suitable for X-ray crystal structure analysis were obtained from the filtered solution over a period of two weeks.

### Refinement

All H atoms were located in a difference Fourier map and refined freely; Csp^2^—H = 0.87 (6) – 0.96 (5) Å, N—H = 0.85 (6) – 0.96 (8) Å and O—H = 0.99 (8) Å.

### Crystal data

**Table:**

C_7_H_8_NO_2_^+^·Cl^−^	*F*(000) = 360
*M**_r_* = 173.59	*D*_x_ = 1.463 Mg m^−^^3^
Monoclinic, *P*2_1_/*c*	Mo *K*α radiation, λ = 0.71073 Å
Hall symbol: -P 2ybc	Cell parameters from 3678 reflections
*a* = 5.601 (5) Å	θ = 2.4–29.3°
*b* = 8.269 (5) Å	µ = 0.43 mm^−^^1^
*c* = 17.118 (5) Å	*T* = 298 K
β = 96.371 (5)°	Block, yellow
*V* = 787.9 (9) Å^3^	0.50 × 0.40 × 0.30 mm
*Z* = 4	

### Data collection

**Table:**

Bruker APEXII CCD area-detector diffractometer	1299 independent reflections
Radiation source: fine-focus sealed tube	1210 reflections with *I* > 2σ(*I*)
graphite	*R*_int_ = 0.030
φ and ω scans	θ_max_ = 25.0°, θ_min_ = 2.7°
Absorption correction: multi-scan (*SADABS*; Bruker, 2008)	*h* = −6→6
*T*_min_ = 0.814, *T*_max_ = 0.882	*k* = −9→7
4205 measured reflections	*l* = −20→20

### Refinement

**Table:**

Refinement on *F*^2^	Secondary atom site location: difference Fourier map
Least-squares matrix: full	Hydrogen site location: inferred from neighbouring sites
*R*[*F*^2^ > 2σ(*F*^2^)] = 0.054	All H-atom parameters refined
*wR*(*F*^2^) = 0.192	*w* = 1/[σ^2^(*F*_o_^2^) + (0.0616*P*)^2^ + 2.6608*P*] where *P* = (*F*_o_^2^ + 2*F*_c_^2^)/3
*S* = 1.26	(Δ/σ)_max_ < 0.001
1299 reflections	Δρ_max_ = 0.49 e Å^−^^3^
133 parameters	Δρ_min_ = −0.34 e Å^−^^3^
0 restraints	Extinction correction: *SHELXL97* (Sheldrick, 2008), Fc^*^=kFc[1+0.001xFc^2^λ^3^/sin(2θ)]^-1/4^
Primary atom site location: structure-invariant direct methods	Extinction coefficient: 0.075 (12)

### Special details

**Table:**

Geometry. All e.s.d.'s (except the e.s.d. in the dihedral angle between two l.s. planes) are estimated using the full covariance matrix. The cell e.s.d.'s are taken into account individually in the estimation of e.s.d.'s in distances, angles and torsion angles; correlations between e.s.d.'s in cell parameters are only used when they are defined by crystal symmetry. An approximate (isotropic) treatment of cell e.s.d.'s is used for estimating e.s.d.'s involving l.s. planes.
Refinement. Refinement of *F*^2^ against ALL reflections. The weighted *R*-factor *wR* and goodness of fit *S* are based on *F*^2^, conventional *R*-factors *R* are based on *F*, with *F* set to zero for negative *F*^2^. The threshold expression of *F*^2^ > σ(*F*^2^) is used only for calculating *R*-factors(gt) *etc*. and is not relevant to the choice of reflections for refinement. *R*-factors based on *F*^2^ are statistically about twice as large as those based on *F*, and *R*- factors based on ALL data will be even larger.

### Fractional atomic coordinates and isotropic or equivalent isotropic displacement parameters (Å^2^)

**Table:**

	*x*	*y*	*z*	*U*_iso_*/*U*_eq_	
C1	0.5758 (9)	0.2824 (5)	0.5157 (3)	0.0322 (11)	
C2	0.3637 (8)	0.1926 (6)	0.4999 (3)	0.0332 (11)	
C3	0.3059 (9)	0.1230 (6)	0.4273 (3)	0.0367 (12)	
C4	0.4607 (8)	0.1434 (5)	0.3701 (3)	0.0314 (11)	
C5	0.6671 (9)	0.2347 (6)	0.3835 (3)	0.0362 (12)	
C6	0.7245 (9)	0.3053 (6)	0.4564 (3)	0.0376 (12)	
C7	0.6379 (9)	0.3487 (6)	0.5964 (3)	0.0348 (11)	
N1	0.4043 (9)	0.0640 (6)	0.2933 (3)	0.0374 (10)	
O1	0.8194 (7)	0.4512 (5)	0.6027 (2)	0.0488 (11)	
O2	0.5313 (8)	0.3078 (5)	0.6508 (2)	0.0573 (12)	
Cl1	0.9009 (2)	0.60977 (15)	0.76491 (7)	0.0407 (5)	
H1	0.858 (13)	0.482 (9)	0.659 (5)	0.09 (2)*	
H1A	0.279 (10)	0.006 (6)	0.293 (3)	0.032 (14)*	
H1B	0.393 (14)	0.131 (11)	0.253 (5)	0.09 (3)*	
H1C	0.545 (14)	0.002 (9)	0.286 (4)	0.08 (2)*	
H2	0.262 (9)	0.183 (6)	0.541 (3)	0.034 (13)*	
H3	0.175 (10)	0.067 (6)	0.419 (3)	0.033 (13)*	
H5	0.780 (10)	0.249 (6)	0.347 (3)	0.042 (15)*	
H6	0.863 (9)	0.363 (6)	0.465 (3)	0.026 (12)*	

### Atomic displacement parameters (Å^2^)

**Table:**

	*U*^11^	*U*^22^	*U*^33^	*U*^12^	*U*^13^	*U*^23^
C1	0.035 (3)	0.030 (2)	0.033 (2)	0.0025 (19)	0.008 (2)	0.0034 (19)
C2	0.028 (2)	0.039 (3)	0.034 (2)	0.001 (2)	0.008 (2)	0.006 (2)
C3	0.033 (3)	0.035 (3)	0.045 (3)	−0.004 (2)	0.013 (2)	0.003 (2)
C4	0.033 (2)	0.028 (2)	0.035 (2)	0.0035 (19)	0.009 (2)	0.0035 (18)
C5	0.035 (3)	0.040 (3)	0.036 (3)	0.000 (2)	0.013 (2)	0.005 (2)
C6	0.033 (3)	0.037 (3)	0.046 (3)	−0.007 (2)	0.015 (2)	−0.001 (2)
C7	0.031 (2)	0.038 (2)	0.034 (2)	0.002 (2)	0.002 (2)	0.003 (2)
N1	0.036 (2)	0.041 (2)	0.035 (2)	−0.004 (2)	0.006 (2)	0.0016 (19)
O1	0.054 (2)	0.054 (2)	0.039 (2)	−0.0211 (19)	0.0078 (19)	−0.0046 (17)
O2	0.059 (3)	0.080 (3)	0.035 (2)	−0.025 (2)	0.014 (2)	−0.0088 (19)
Cl1	0.0353 (8)	0.0408 (8)	0.0459 (8)	0.0035 (5)	0.0043 (5)	−0.0016 (5)

### Geometric parameters (Å, °)

**Table:**

C1—C6	1.395 (6)	C5—C6	1.383 (7)
C1—C2	1.402 (7)	C5—H5	0.94 (5)
C1—C7	1.491 (7)	C6—H6	0.91 (5)
C2—C3	1.374 (7)	C7—O2	1.209 (6)
C2—H2	0.96 (5)	C7—O1	1.319 (6)
C3—C4	1.389 (6)	N1—H1A	0.85 (6)
C3—H3	0.87 (6)	N1—H1B	0.88 (9)
C4—C5	1.378 (7)	N1—H1C	0.96 (8)
C4—N1	1.471 (7)	O1—H1	0.99 (8)
			
C6—C1—C2	119.6 (5)	C6—C5—H5	116 (3)
C6—C1—C7	121.8 (5)	C5—C6—C1	120.1 (5)
C2—C1—C7	118.6 (4)	C5—C6—H6	118 (3)
C3—C2—C1	120.4 (4)	C1—C6—H6	121 (3)
C3—C2—H2	122 (3)	O2—C7—O1	124.0 (5)
C1—C2—H2	117 (3)	O2—C7—C1	121.8 (5)
C2—C3—C4	119.0 (5)	O1—C7—C1	114.2 (4)
C2—C3—H3	118 (3)	C4—N1—H1A	111 (3)
C4—C3—H3	123 (3)	C4—N1—H1B	114 (5)
C5—C4—C3	121.7 (5)	H1A—N1—H1B	111 (6)
C5—C4—N1	119.1 (4)	C4—N1—H1C	104 (5)
C3—C4—N1	119.1 (4)	H1A—N1—H1C	113 (6)
C4—C5—C6	119.3 (4)	H1B—N1—H1C	103 (6)
C4—C5—H5	125 (3)	C7—O1—H1	109 (4)
			
C6—C1—C2—C3	2.1 (7)	C4—C5—C6—C1	0.7 (8)
C7—C1—C2—C3	−176.9 (4)	C2—C1—C6—C5	−2.3 (7)
C1—C2—C3—C4	−0.1 (7)	C7—C1—C6—C5	176.6 (5)
C2—C3—C4—C5	−1.6 (7)	C6—C1—C7—O2	−167.9 (5)
C2—C3—C4—N1	177.7 (4)	C2—C1—C7—O2	11.1 (7)
C3—C4—C5—C6	1.4 (7)	C6—C1—C7—O1	10.8 (7)
N1—C4—C5—C6	−177.9 (5)	C2—C1—C7—O1	−170.2 (4)

### Hydrogen-bond geometry (Å, °)

**Table:**

*D*—H···*A*	*D*—H	H···*A*	*D*···*A*	*D*—H···*A*
O1—H1···Cl1	0.99 (8)	2.10 (8)	3.059 (4)	164 (6)
N1—H1A···Cl1^i^	0.85 (6)	2.33 (6)	3.154 (6)	165 (5)
N1—H1B···O2^ii^	0.88 (9)	2.05 (8)	2.823 (6)	145 (7)
N1—H1B···Cl1^iii^	0.88 (9)	2.70 (9)	3.289 (5)	125 (6)
N1—H1C···Cl1^ii^	0.96 (8)	2.26 (8)	3.215 (5)	172 (6)

Symmetry codes: (i) *x*−1, −*y*+1/2, *z*−1/2; (ii) *x*, −*y*+1/2, *z*−1/2; (iii) −*x*+1, −*y*+1, −*z*+1.
